# Trends and Themes in the Study of Value in Orthopedic Surgery: A Systematic Review

**DOI:** 10.1177/15563316231204040

**Published:** 2023-10-24

**Authors:** Hassaan Abdel Khalik, Manraj S. Nijjar, Jack Soeder, Darius L. Lameire, Herman Johal

**Affiliations:** 1 Division of Orthopaedic Surgery, McMaster University, Hamilton, ON, Canada; 2 Michael G. DeGroote School of Medicine, McMaster University, Hamilton, ON, Canada; 3 Division of Orthopaedic Surgery, Department of Surgery, University of Toronto, Toronto, ON, Canada

**Keywords:** value-based care, cost-effectiveness analysis, cost-utility analysis, health economics

## Abstract

**Background:**

The study of value in orthopedic surgery aims to maximize health outcomes gained per unit cost through various health economic tools but is fragmented across various subspecialties and geographies. Therefore, it is difficult to ascertain whether this research methodology is being used to its full potential across all orthopedic subspecialties and geographies.

**Purpose:**

We sought to assess the distribution of prior health economics literature in orthopedic surgery across subspecialties and geographies. The secondary aim was to identify pertinent methodologic trends that may affect the conclusions drawn.

**Methods:**

A systematic review utilizing 3 electronic databases (Medline, Embase, and Web of Science) was performed. Inclusion criteria included prior systematic reviews assessing economic analyses across all orthopedic surgery subspecialities published between 2010 and April 24, 2021. The quality of evidence was assessed using the Assessment of Multiple Systematic Review tool. Data were qualitatively analyzed.

**Results:**

In the 44 studies included, arthroplasty (36.4%) and spine (31.8%) were the most represented subspecialties. Almost half of studies originated from the United States (45.5%), followed by the United Kingdom (18.2%). Health economic models were most commonly from the perspective of the health care or hospital system (40.5%), followed by the societal perspective (23.5%), and the payer perspective (14.8%).

**Conclusions:**

The study of value in orthopedic surgery is not uniformly leveraged across all subspecialties and geographies. Methodologically, the societal perspective was inadequately represented, despite orthopedic pathologies often incurring significant indirect costs (eg, time off work, rehabilitation expenses).

## Introduction

Orthopedic conditions pose a significant economic burden on health care systems worldwide. In the United States alone, annual spending on musculoskeletal conditions has been estimated to be $380 billion, approximately 14% of overall health care spending [19]. Evaluating value in orthopedic surgery is a growing research field. The study of value in orthopedic surgery aims to maximize the health outcomes gained per unit cost [46].

Health economic evaluations are powerful tools that can be used to quantify the value of novel interventions in orthopedic surgery [7]. Health economic evaluations utilize a highly modular study design that allows researchers to answer very specific questions pertaining to the interventions being evaluated, the model of care in which an intervention is being implemented, and the availability of data that can be used in the model.

First, health economic studies can comprise several designs, including, but not limited to, cost-effectiveness, cost-utility, cost-description, cost-minimization, and cost-benefit analyses [64]. Second, researchers can decide on the perspective of their study (ie, health care system, hospital, or payer) [64]. This allows for stakeholder-relevant conclusions regarding the value of a novel intervention. Third, researchers must decide on whether they want to base their evaluation on a preexisting study, also known as a trial-based study, or develop a model that draws on several data sources, also known as a model-based study [64]. Additional modifiable study-specific characteristics include the country on which a model is based, the number of interventions being evaluated, and the time horizon of the study. Studies evaluating value in orthopedic surgery have been found to be highly heterogeneous, rendering it difficult to consistently build upon previous published works [38].

The current literature on value in orthopedic surgery is fragmented. Various systematic reviews have been published with the aims of aggregating health economics literature in orthopedic surgery. These reviews predominantly focused on either the quality of the literature [8,16,34,48] or the overarching cost-effectiveness of an intervention [10,11,44]. Although these reviews are helpful when drawing conclusions specific to an orthopedic intervention or subspecialty, it remains difficult to draw conclusions about the study of value in orthopedic surgery as a whole. Furthermore, the heterogeneous nature of these systematic reviews renders it difficult to identify which orthopedic subspecialties and interventions have been thoroughly evaluated through the lens of health economic evaluations and which have not. A possible solution to the rapidly growing yet fragmented body of literature in this field is to conduct a systematic review of reviews [63].

The primary aim of this study was to identify existing systematic reviews of health economics literature in orthopedic surgery and assess their distribution across orthopedic subspecialty and country of origin. The secondary aim was to identify methodologic trends in those studies, including the type of health economic model utilized, whether the model was trial-based or model-based, the perspectives of the models, and the time horizons implemented.

## Methods

A systematic review was conducted of published systematic reviews relating to economic analysis in orthopedic surgery. This review followed the guidelines and algorithm of the Preferred Reporting Items for Systematic Reviews and Meta-Analyses [41]. A systematic search of Medline, Embase, and Web of Science was performed, retrieving articles published between January 1, 2010, and April 24, 2022. The search terms included “orthopedic surgery,” “cost,” and “systematic review.” The complete search strategies used can be found in Supplemental Table 1.

Inclusion criteria for this review were systematic reviews with a focus on orthopedic surgery (all orthopedic subspecialties) and economic analyses (cost-utility analysis, cost-effectiveness analysis, cost-minimization analysis, cost-benefit analysis, and cost-description analysis). Exclusion criteria included non-English articles, nonorthopedic surgery studies, studies with an aim that was not exclusive to health economic evaluations, health technology assessment articles, inadequate (less than 3) health economic studies included in systematic review, inadequately reported outcomes, and publication prior to 2010.

The titles and abstracts of the identified studies were independently screened by 2 authors (J.S. and M.S.N.) and those not meeting inclusion criteria were discarded. Disagreements at this stage were advanced to the full-text review stage to prevent any premature exclusions. The full texts of the remaining articles were then retrieved and reviewed against the inclusion and exclusion criteria by 2 authors (J.S. and M.S.N.). Disagreements in this full-text review stage were resolved by consulting a third, senior author (H.A.K.).

The strength and quality of evidence in the included studies were assessed by 4 authors (H.A.K., D.L.L., J.S., and M.S.N.) using the Assessment of Multiple Systematic Reviews (AMSTAR) instrument, a validated quality assessment tool for systematic reviews [62]. The tool includes 11 questions addressing items such as study design, conflict of interest, and publication bias. A 4-point scoring scale was used to quantify the performance of the included reviews against each domain, as performed in previous economic analysis publications [18,23,68]. As described in Diaby et al [18], the 4-point response choices of yes, no, and can’t answer, were assigned the scores of 1, 0, and 0, respectively. For nonapplicable domains, the maximum score was reduced by 1 to allow for comparison between studies. This new scoring scale was used to adapt the existing AMSTAR tool to fit the needs of our study, specifically, by expressing final scores as percentages to allow for comparison of study quality across the included systematic reviews.

Four reviewers (H.A.K., D.L.L., J.S., and M.S.N.) each abstracted data from a quarter of the included studies. The data were abstracted and recorded into predetermined tables, using Google Sheets. The following data were abstracted from the studies, if available: study characteristics (author, journal, publication year, subspecialty, study type, and country); study objectives, primary findings, and type of economic evaluation (eg, cost-utility analysis, cost-effectiveness analysis); health economic study quality assessment tool used; quality of evidence reported by review; perspectives of economic evaluations; and time horizons.

A qualitative summary analysis was performed based on this review’s primary and secondary objectives. Percent distributions and weighted means were calculated based on various study characteristics.

## Results

**Fig. 1.**
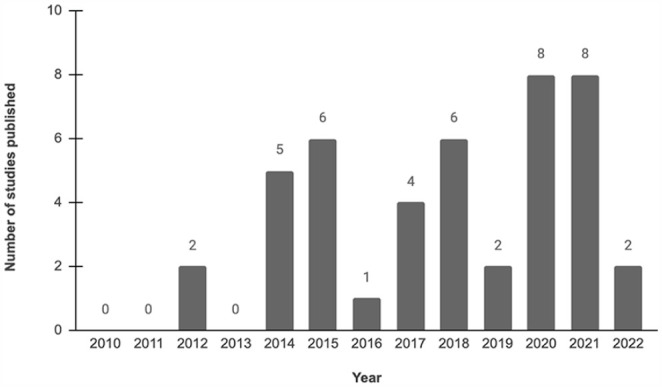
Studies by publication year, retrieving articles between January 1, 2010 and April 24, 2022. The year 2022 only comprises studies up until search strategy end date of April 24, 2022.

Our search identified 8324 studies, with 6694 remaining after the exclusion of duplicates (Supplemental Figure 1). Forty-four studies were eligible for this review (Supplemental Table 2) [1–5,9–17,20,21,25,27,28,30–33,35,37,39,40,42–45,47,49–52,54–56,58–60,65,66]. The average AMSTAR scores for included studies was 71%, or relatively good quality (Supplemental Table 2). A mean of 19 studies (range, 4–52) were included per systematic review. The rate of reviews published over this study’s inclusion period (2010 to April 2022) was constant, with an uptick in 2020 (Fig. 1).

**Fig. 2.**
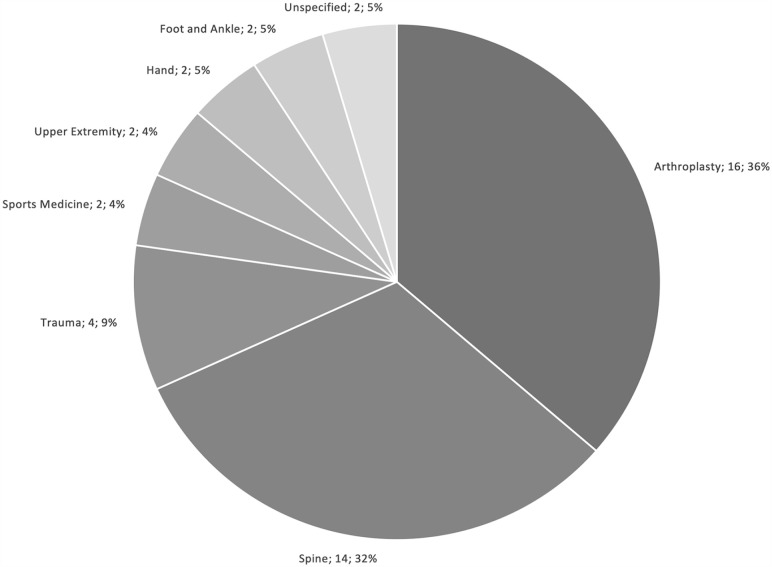
Studies by subspecialty.

**Fig. 3.**
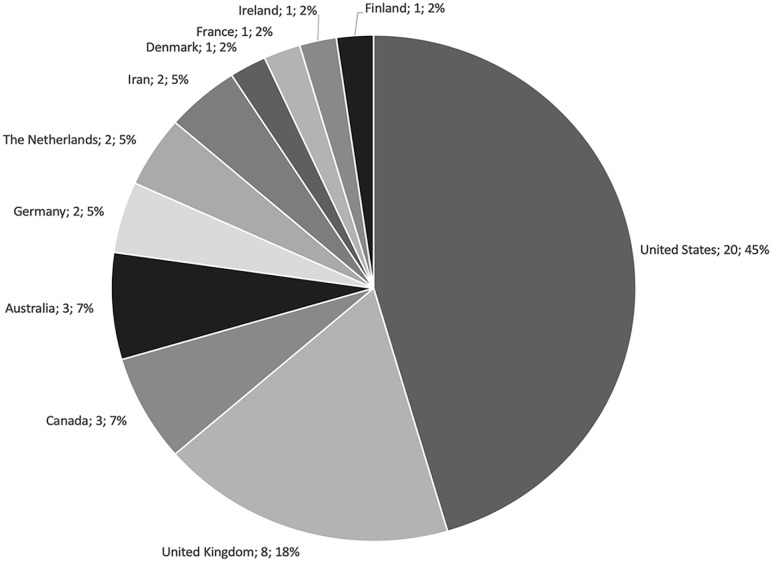
Studies by country of origin.

Arthroplasty and spine were the most studied subspecialties, representing 36.4% and 31.8% of included studies, respectively (Fig. 2). Almost half of the included studies (45.5%) originated from research groups in the United States, followed by 18.2% from the United Kingdom, and 6.8% each from Canada and Australia (Fig. 3).

More than two thirds of systematic reviews conducted a quality assessment of their included studies (70.5%). The Quality of Health Economics Studies checklist was the most utilized quality assessment tool, followed by the Consolidated Health Economic Evaluation Reporting Standards and Consensus Health Economic Criteria (Supplemental Figure 2).

Cost-utility analyses were the most utilized health economic methodology, comprising over half of the studies included in eligible systematic reviews (55.0%) (Supplemental Table 3). Cost-effectiveness analyses were the second most common methodology utilized, comprising 23.7% of included studies. This trend generally persisted across all subspecialties. Trauma had a noticeably different distribution of health economic study designs, with cost-utility analyses and cost-description analyses comprising an equal percentage of studies (34.7% and 37.3%, respectively).

Studies from the perspective of the health care or hospital system were the most common, comprising almost half of the included studies (40.5%), followed by the societal perspective (23.5%) and the payer perspective (14.8%; Supplemental Table 3). Exceptions to this trend included trauma surgery (where the most common perspective was the payer’s [69.1%]) and spine surgery (where both the health care/hospital perspective and the societal perspective were evenly distributed [36.7% and 30.6%, respectively]). A minority of studies conducted their analysis from multiple perspectives (8.4%).

Over half of included studies were trial based (53.3%) and 36.6% were model based. The structure was unclear or unknown in 10.6%.

The most utilized time horizon across all subspecialties was from 0 to 5 years (39.4%), followed by the lifetime horizon (14.5%). The remaining time horizons were evenly distributed between 5% and 6% each. Notably, 27.6% of systematic reviews did not report the time horizons of their included studies. When eliminating studies with unknown time horizons, more than half of the studies utilized the time horizon of 0 to 5 years (54.4%), followed by 20% modeling their outcomes to a lifetime horizon. Arthroplasty (30.8%), foot and ankle (50.0%), sports medicine (41.5%), and upper extremity (24.3%) had a significant proportion of their models using a lifetime horizon.

## Discussion

With the economic burden of musculoskeletal pathology only projected to grow worldwide [26], it is imperative that orthopedic surgeons seek out both clinically and cost-effective treatments. The study of value in orthopedic surgery aims to shed light on the most cost-effective treatment options for various pathologies [46]. Through health economic modeling, researchers can assess the quality of life attained per unit cost for a given intervention and compare this ratio with the current standard of care.

Although this research field has grown, it is still nascent and may not be familiar to orthopedic surgeons without a background in health economics research. Thus, several systematic reviews have attempted to amalgamate the findings of health economic literature across various orthopedic subspecialties. Nonetheless, to our knowledge, no study has attempted to amalgamate all the health economic literature in orthopedic surgery to identify key trends that could guide future directions in the field. Therefore, the purpose of this systematic review of reviews was to elucidate key insights on the state of value research in orthopedic surgery and recommendations for future research.

Key findings of this study were the following: (1) arthroplasty (36.4%) and spine (31.8%) were the 2 most commonly evaluated subspecialties; (2) almost half of published reviews were from the United States (45.6%); (3) cost-utility analyses (55.0%) and cost-effectiveness analyses (23.7%) were the most commonly utilized health economic models; (4) the health care or hospital perspective (40.5%) was the most common perspective of models, almost double the next most common perspective (societal, 23.5%); (5) more than half of the models were trial based (53.3%); and (6) the time horizon of zero to 5 years was the most commonly implemented horizon (39.4%). Ultimately, our findings suggest that health economic modeling in orthopedic surgery is underrepresented in several subspecialties, with disproportionate interest from the United States.

As with any review, this study is not without limitations. First, only studies published since 2010 were included to ensure the relevance of this study’s findings. Second, the conclusions drawn from this study are based on previous systematic reviews, not individual studies, and therefore studies in various underrepresented subspecialties may not be accounted for. Nonetheless, systematic reviews can be viewed as a proxy for the breadth and recency of published literature in a field. Third, this study’s abstracted data were based on the reporting of previous reviews and, consequently, relied on the quality of included reviews. Based on the AMSTAR quality assessment tool, the quality of included studies was good; therefore, the findings of this review can be interpreted with confidence.

Arthroplasty (36.4%) and spine (31.8%) comprised the majority of included reviews, followed by trauma (9.1%). Several epidemiologic and surgical factors drive this trend. According to the World Health Organization, osteoarthritis, low-back pain, and fragility fractures secondary to osteoporosis are the 3 most common disability musculoskeletal pathologies [67]. As a result, spine surgery and arthroplasty are 2 high-volume specialties that are projected to grow due to an aging population. Both subspecialties are also associated with adverse sequelae that are catastrophic from both clinical and cost perspectives. Revision joint arthroplasty, which poses high morbidity for the patient, is projected to grow due to several factors, including patients outliving their prostheses [24], an increasingly obese and infection-prone population undergoing the surgery [22], and periprosthetic fractures [53]. Similarly, revision spine surgery is also projected to grow although its proportion to index surgeries may be decreasing [57]. In addition, unlike sports and hand procedures, which can be done in an ambulatory surgical center, arthroplasty and spine surgeries generally require postoperative admission, further increasing costs [36]. Therefore, the overrepresentation of arthroplasty, spine surgery, and trauma surgery may be justified when considering the prevalence of, and disability associated with, their respective pathologies. Nonetheless, clinicians and researchers from the remaining orthopedic subspecialties are encouraged to utilize health economic analyses to inform practice and policy, especially when considering that resources are ultimately shared.

The generalizability of health economic evaluations is limited to the health care model in which they are conducted. A recent study categorized Organization for Economic Co-operation and Development country health care systems into 5 categories: the National Health Service, the National Health Insurance, the Societal Health Insurance, the Etatist Social Health Insurance, and the Private Health System [6]. When the country of origin of included reviews was categorized based on this classification system, the Private Health System comprised almost half of the published reviews (48%), followed by the National Health Service (24%), National Health Insurance (17%), the Etatist Social Health Insurance (7%), and the Social Health Insurance (5%). This trend was driven by a majority of reviews being published in the United States, a Private Health System [6]. Furthermore, the United States was the only Organization for Economic Co-operation and Development health care system categorized as a Private Health System. This calls into question the international generalizability of health economic evaluations in orthopedic surgery. Research groups based in countries with differing health care models are encouraged to produce health economic evaluations from their perspective to broaden this research area’s diversity of perspectives.

The perspective from which a health economic evaluation is carried out dictates which stakeholders would find the model’s conclusions relevant. Possible perspectives include societal, health care or hospital system, or payer. The societal perspective is the most difficult to account for although it is arguably the most informative [29]. This is reflected by the second panel’s recommendation to consider both health care payer and societal perspective when conducting health economic evaluations [61]. Furthermore, when considering that the objective of most orthopedic surgeries is to restore function and ability, not assessing the fiscal implications of such objectives ignores a possibly significant benefit provided by orthopedic interventions. This review demonstrated that only 23.5% of included studies conducted their analysis from the societal perspective, with spine surgery reporting this perspective most often (30.6%). Therefore, future health economic evaluations in orthopedic surgery should make a concerted effort to include the societal perspective in their analysis.

In summary, this systematic review found that health economic evaluations in orthopedic surgery are reflective of the pathologies responsible for the highest economic burden. Specifically, most reviews focused on either arthroplasty or spine surgery, followed by trauma. Two major areas of improvement were also identified. First, not all health care systems were represented equally, limiting the generalizability of findings. Second, the societal perspective was undertaken only 23.5% of the time, despite it being informative to the greatest number of stakeholders. The societal perspective is also especially relevant to orthopedic surgery, where the objective is to restore function and ability, which could have a ripple effect of positive economic benefits (eg, patients being able to return to work). Recommendations for future research include (1) publishing health economic evaluations by research groups from a diverse array of health care systems, and (2) conducting health economic evaluations wherein the societal perspective is also accounted for.

## Supplemental Material

sj-docx-1-hss-10.1177_15563316231204040 – Supplemental material for Trends and Themes in the Study of Value in Orthopedic Surgery: A Systematic Review Supplemental material, sj-docx-1-hss-10.1177_15563316231204040 for Trends and Themes in the Study of Value in Orthopedic Surgery: A Systematic Review by Hassaan Abdel Khalik, Manraj S. Nijjar, Jack Soeder, Darius L. Lameire and Herman Johal in HSS Journal®

sj-docx-2-hss-10.1177_15563316231204040 – Supplemental material for Trends and Themes in the Study of Value in Orthopedic Surgery: A Systematic Review Supplemental material, sj-docx-2-hss-10.1177_15563316231204040 for Trends and Themes in the Study of Value in Orthopedic Surgery: A Systematic Review by Hassaan Abdel Khalik, Manraj S. Nijjar, Jack Soeder, Darius L. Lameire and Herman Johal in HSS Journal®

sj-docx-3-hss-10.1177_15563316231204040 – Supplemental material for Trends and Themes in the Study of Value in Orthopedic Surgery: A Systematic Review Supplemental material, sj-docx-3-hss-10.1177_15563316231204040 for Trends and Themes in the Study of Value in Orthopedic Surgery: A Systematic Review by Hassaan Abdel Khalik, Manraj S. Nijjar, Jack Soeder, Darius L. Lameire and Herman Johal in HSS Journal®

sj-docx-4-hss-10.1177_15563316231204040 – Supplemental material for Trends and Themes in the Study of Value in Orthopedic Surgery: A Systematic Review Supplemental material, sj-docx-4-hss-10.1177_15563316231204040 for Trends and Themes in the Study of Value in Orthopedic Surgery: A Systematic Review by Hassaan Abdel Khalik, Manraj S. Nijjar, Jack Soeder, Darius L. Lameire and Herman Johal in HSS Journal®

sj-pdf-7-hss-10.1177_15563316231204040 – Supplemental material for Trends and Themes in the Study of Value in Orthopedic Surgery: A Systematic Review Supplemental material, sj-pdf-7-hss-10.1177_15563316231204040 for Trends and Themes in the Study of Value in Orthopedic Surgery: A Systematic Review by Hassaan Abdel Khalik, Manraj S. Nijjar, Jack Soeder, Darius L. Lameire and Herman Johal in HSS Journal®

sj-tiff-5-hss-10.1177_15563316231204040 – Supplemental material for Trends and Themes in the Study of Value in Orthopedic Surgery: A Systematic Review Supplemental material, sj-tiff-5-hss-10.1177_15563316231204040 for Trends and Themes in the Study of Value in Orthopedic Surgery: A Systematic Review by Hassaan Abdel Khalik, Manraj S. Nijjar, Jack Soeder, Darius L. Lameire and Herman Johal in HSS Journal®

sj-tiff-6-hss-10.1177_15563316231204040 – Supplemental material for Trends and Themes in the Study of Value in Orthopedic Surgery: A Systematic Review Supplemental material, sj-tiff-6-hss-10.1177_15563316231204040 for Trends and Themes in the Study of Value in Orthopedic Surgery: A Systematic Review by Hassaan Abdel Khalik, Manraj S. Nijjar, Jack Soeder, Darius L. Lameire and Herman Johal in HSS Journal®
