# Supplementary material for: Trends and Themes in the Study of Value in Orthopedic Surgery: A Systematic Review
Source: HSS J. 2023 Oct 24;21(1):93–101. doi: 10.1177/15563316231204040 (PMC11748386; doi:10.1177/15563316231204040)
Supplement: sj-docx-1-hss-10.1177_15563316231204040 – Supplemental material for Trends and Themes in the Study of Value in Orthopedic Surgery: A Systematic Review [file sj-docx-1-hss-10.1177_15563316231204040.docx]

**Supplemental Table 1.** The complete search strategies used to conduct a systematic review of published systematic reviews relating to economic analysis in orthopedic surgery.

| **Medline** | **Embase** | **Web of Science** |
| --- | --- | --- |
| 1. exp Orthopedic Procedures/ or orthopaedic procedure.mp. 2. orthopedic surgery.mp. 3. exp Orthopedics/ 4. exp Fractures, Bone/ 5. fracture.mp. 6. arthroscopy.mp. or exp Arthroscopy/ 7. exp Arthroplasty/ or arthroplasty.mp. 8. exp "Costs and Cost Analysis"/ 9. cost.mp. 10. exp "Health Care Economics and Organizations"/ 11. exp "Delivery of Health Care"/ 12. value-based.mp. 13. exp "Systematic Review"/ 14. systematic review.mp. 15. review.mp. 16. 1 or 2 or 3 or 4 or 5 or 6 or 7 17. 8 or 9 or 10 or 11 or 12 18. 13 or 14 or 15 19. 16 and 17 and 18 20. limit 19 to english language 21. limit 20 to humans 22. limit 21 to yr="2010 -Current" | 1. exp orthopedic surgery/ 2. orthopedic procedures.mp. 3. orthopedic surgery.mp. 4. exp fracture/ 5. fracture*.mp. 6. arthroscopy.mp. or exp arthroscopy/ 7. exp arthroplasty/ or arthroplasty.mp. 8. exp "cost benefit analysis"/ 9. cost.mp. 10. exp "health care cost"/ 11. value-based.mp. 12. exp "systematic review"/ 13. systematic review.mp. 14. review.mp. 15. 1 or 2 or 3 or 4 or 5 or 6 or 7 16. 8 or 9 or 10 or 11 17. 12 or 13 or 14 18. 15 and 16 and 17 19. limit 18 to english language 20. limit 19 to human 21. limit 20 to yr="2010 -Current" | (TS=orthop*edic OR TS=orthop*edic surger* OR TS=orthop*edic procedur*) AND (TS=cost OR TS=value-based care) AND (TS=systematic review OR TS=meta-analysis OR TS=metaanalysis) |
