# Supplementary material for: Trends and Themes in the Study of Value in Orthopedic Surgery: A Systematic Review
Source: HSS J. 2023 Oct 24;21(1):93–101. doi: 10.1177/15563316231204040 (PMC11748386; doi:10.1177/15563316231204040)
Supplement: sj-docx-2-hss-10.1177_15563316231204040 – Supplemental material for Trends and Themes in the Study of Value in Orthopedic Surgery: A Systematic Review [file sj-docx-2-hss-10.1177_15563316231204040.docx]

**Supplemental Table 2** Study Overview

| **Author** | **Year Published** | **Sub-Specialty** | **Country** | **Review Period** | **Aim/Objective as per Study*** | **AMSTAR Score, %** |
| --- | --- | --- | --- | --- | --- | --- |
| Afzali et al [1] | 2018 | Unspecified | Denmark | Inception to 2017 | Identify, gather, and appraise studies reporting on the cost-effectiveness of different treatment approaches for nonosteoarthritic knee pain conditions in adolescents and adults to identify cost-effective treatments. | 90% |
| Agarwal et al [2] | 2021 | Arthroplasty | United Kingdom | 1997 to 2019 | Evaluate the international quality and breadth of the current cost-effectiveness analyses conducted on total hip arthroplasty in the management in hip osteoarthritis. | 80% |
| Alvin et al [3] | 2014 | Spine | United States | 1976 to 2014 | Systematically review all cost-effectiveness studies in spine surgery literature, identify how costs were defined and calculated, and compare cost methodologies among studies analyzing the same intervention. | 50% |
| Alvin et al [4] | 2014 | Spine | United States | Inception to 2014 | Perform an evidence-based synthesis of the literature, assessing the cost-effectiveness of surgical intervention for syndromes associated with cervical degenerative disc disease. | 90% |
| Bielska et al [5] | 2019 | Trauma | Canada | 1980 to 2014 | Collect, assess, and critically appraise the published literature on the health economics of ankle and foot injury treatment. | 73% |
| Brockbank and Wolawacz [9] | 2017 | Arthroplasty | United Kingdom | 2008 to 2015 | Identify published economic analyses of NOACs for primary VTE prophylaxis following THA and TKA surgeries, and to summarize the modelling techniques used and the cost-effectiveness results. | 50% |
| Burn at al [10] | 2017 | Arthroplasty | United Kingdom | Not specified | Identify economic evaluations comparing UKAs and TKAs, examine approaches utilized, assess the quality of these evaluations, and consider implications of value. | 80% |
| Büttner et al [11] | 2020 | Arthroplasty | Germany | 2007 to 2017 | Summarize and analyze the current state of research regarding costs of hip or knee arthroplasty following a fast-track protocol and to evaluate whether a cost reduction can be achieved for patients undergoing a multidisciplinary fast-track treatment instead of classic knee or hip arthroplasty. | 70% |
| Caelers et al [12] | 2021 | Spine | The Netherlands | Inception to 2020 | Compare economic evaluations of TLIF and PLIF, directly and indirectly, in adults with lumbar spondylosthesis while assessing the methodologic quality of included studies. | 100% |
| Chang et al [13] | 2020 | Spine | United States | Inception to 2019 | Systematically review all cost-effectiveness studies reported on spine surgery, highlighting the strengths and weaknesses of these CEAs. | 90% |
| Chawla et al [14] | 2016 | Arthroplasty | United States | Inception to 2016 | Synthesize findings from existing CEA studies to reach generalizable and actionable UKA implant survivorship goals from an economic perspective. | 100% |
| Coyle et al [15] | 2018 | Trauma | Ireland | 1990 to 2015 | Systematically review the CUA evidence to date on interventions in orthopaedic trauma care. | 70% |
| Cregar et al [16] | 2021 | Arthroplasty | United States | Not specified | Provide a comprehensive review of published United States-based cost analyses pertaining to shoulder arthroplasty and to evaluate the overall quality of available studies using a validated instrument and identify factors predictive of the achievement of low- and high-quality scores. | 80% |
| Daigle et al [17] | 2012 | Arthroplasty | United States | January 1, 1980, to February 1, 2012 | Summarize the state of the literature evaluating the cost-effectiveness of elective total hip and knee arthroplasty. | 50% |
| Dritsaki et al [20] | 2018 | Hand | United Kingdom | January 1996 to November 2016 | Conduct a systematic literature review to identify the extent and appraise the quality of the existing literature on the cost-effectiveness of treatments for Dupuytren's disease. | 90% |
| Droeghaag et al [21] | 2021 | Spine | The Netherlands | Inception to April 3, 2020 | Evaluate the current literature on cost-effectiveness of open transforaminal lumbar interbody fusion compared to minimally invasive transforaminal lumbar interbody fusion in patients with lumbar spondylolisthesis or degenerative disease. | 82% |
| Harrop et al [25] | 2014 | Spine | United States | Inception to December 16, 2013 | Review the available evidence on the cost-effectiveness of spinal surgery for 2 common lumbar conditions–degenerative spondylolisthesis and spinal stenosis without instability or deformity. | 60% |
| Hsu et al [27] | 2014 | Spine | United States | Inception to December 2013 | Provide a systematic review of the literature on bone graft substitutes and extenders with the intention of addressing the cost-effectiveness of bone graft extenders and substitutes in spine surgery. | 60% |
| Indrakanti et al [28] | 2012 | Spine | United States | Inception to June 24, 2010 | Systematically review the literature on cost-utility analysis for the management of symptomatic low back pain. | 70% |
| Kamaraj et al [30] | 2020 | Arthroplasty | United Kingdom | January 1, 1997 to February 2, 2020 | Assess the scope and quality of all current TKA cost-effectiveness analysis studies. | 80% |
| Kamaruzman et al [31] | 2017 | Arthroplasty | United Kingdom | 2004 to 2016 | Review published economic evaluations aimed at assessing and summarizing evidence on the cost-effectiveness of surgical interventions for the management of osteoarthritis. | 80% |
| Karhade et al [32] | 2018 | Foot and Ankle | United States | January 2000 to December 2017 | Conduct a systematic review of the orthopaedic foot and ankle cost-utility literature to provide the practicing orthopaedic surgeon with a critical understanding of the literature’s strengths, weaknesses, and areas for future improvement. | 30% |
| Kask et al [33] | 2020 | Trauma | Finland | Inception to March 9, 2020 | Investigate the cost-effectiveness of operative and nonoperative clavicle fracture treatment. | 80% |
| Lan et al [35] | 2020 | Arthroplasty | United States | January 1, 2005 to April 8, 2020 | Assess and compare the cost-effectiveness of THA and TKA via economic analyses, based on the published literature | 60% |
| Lubelski et al [37] | 2014 | Spine | United States | Inception to Dec 24, 2013 | Review the economic literature evaluating the cost-effectiveness of minimal access surgery compared with conventional open procedures for the cervical and lumbar spine. | 80% |
| Martelli et al [39] | 2015 | Spine | France | Inception to March 2014 | Assess the level of evidence provided by the available economic evaluations for vertebroplasty and kyphoplasty. | 70% |
| McAnany et al [40] | 2015 | Spine | United States | January 1980 to June 2014 | Provide an overview of the types of decision analytic models used in spine surgery and provide a systematic overview of the most cited studies in the spine literature. | 50% |
| Mordin et al [42] | 2021 | Arthroplasty | United States | Jan 2000 to Oct 2020 | Review the literature to determine the economic impact of intra-articular hyaluronic acid for pain associated with knee osteoarthritis in the United States. | 60% |
| Nolte et al [43] | 2019 | Hand | United States | 1990 to not specified | Review the literature on orthopedic surgical trips. Analyze the cost-effectiveness of efforts to train and educate orthopedic surgeons in LMICs, a growing area of outreach interest. Determine the degree of cost-effectiveness according to WHO-CHOICE thresholds and to examine the specific type of orthopedic interventions being offered. | 80% |
| Nwachukwu et al [44] | 2015 | Arthroplasty | United States | January 1999 to January 2014 | Determine the cost utility of TJA interventions; critically assess the quality of published US-based cost utility analyses using the Quality of Health Economic Studies instrument; and determine what characteristics were common among studies receiving a high-quality score. | 80% |
| Nwachukwu et al [47] | 2015 | Sports Medicine | United States | January 1998 to January 2014 | Identify and summarize CEA studies in orthopaedic sports medicine and to grade the quality of the available literature. | 80% |
| Nwachukwu et al [49] | 2015 | Spine | United States | January 1999 to January 2014 | Identify United States-based cost-utility analyses in spine care and to critically evaluate the quality of the available literature. | 80% |
| Nwachukwu et al [45] | 2018 | Unspecified | United States | Inception to April 2017 | Identify previously published CVM studies in the orthopaedic literature, assess the methodologies used for CVM research, and understand how CVM has been used in the orthopaedic cost-benefit analysis framework. | 50% |
| Okafor et al [50] | 2021 | Arthroplasty | Australia | Inception to November 23, 2020 | Review the literature on the cost of revision TKA for septic and aseptic causes and to identify the major cost components contributing to the cost burden. | 80% |
| Parkes et al [51] | 2017 | Trauma | United Kingdom | Not specified | Evaluate the cost-effectiveness of moving from traction to intramedullary nailing for femoral shaft fractures in adults in less economically developed countries. | 60% |
| Phan et al [52] | 2015 | Spine | Australia | Inception to January 2015 | Identify all relevant studies comparing cost-utility outcomes specifically for minimally invasive-TLIF versus open-TLIF, excluding other spine surgery procedures, compare direct hospital costs statistically using meta-analysis techniques, and identify potential perioperative factors driving any differences in costs. | 70% |
| Primeau et al [54] | 2021 | Sports Medicine | Canada | Inception to March 1, 2020 | Evaluate and summarize reporting of results in the orthopaedic sports medicine literature on cost-effectiveness for knee and hip interventions, assess the quality of published full economic evaluations using the QHES tool, and identify areas in which study quality improvement is required. | 82% |
| Pritchard et al [55] | 2020 | Arthroplasty | United Kingdom | January 2000 to August 2019 | Assess cost-effectiveness of enhanced recovery pathways following total hip and knee arthroplasties, report on quality of studies, and identify research gaps for future work | 80% |
| Pron et al [56] | 2022 | Spine | Canada | Inception to May 2021 | Provide an up-to-date summary and comparison of methodologies and results of peer-reviewed health economic studies used to evaluate cost-effectiveness of vertebroplasty and balloon kyphoplasty treatment of osteoporotic vertebral fractures (OVFs) to support treatment and health care funding decisions. | 60% |
| Rajan et al [58] | 2018 | Upper Extremity | United States | January 1, 1997 to December 2016 | Assess the quality and scope of the current CEA literature in the field of hand and upper limb orthopaedic surgery. | 50% |
| Rashki Kemmak et al [59] | 2020 | Arthroplasty | Iran | 2007 to 2009 | Investigate the published cost-effectiveness evidence of rivaroxaban versus enoxaparin for the prevention of VTE after TKA and THA. | 50% |
| Rezapour et al [60] | 2021 | Arthroplasty | Iran | January 2008 to December 2019 | Summarize evidence from economic evaluations regarding NOACs used in VTE prophylaxis after joint replacement surgery. | 70% |
| Tischer et al [65] | 2020 | Sports Medicine | Germany | Inception to January 1, 2010 | Analyze the current state of cost-effectiveness analyses in shoulder surgery. | 60% |
| Walsh et al [66] | 2022 | Foot and Ankle | Australia | Inception to January 13, 2020 | Identify, appraise, and synthesize the literature pertaining to the cost-effectiveness of interventions for musculoskeletal foot and ankle conditions. | 70% |

*Language used for study objectives has been minimally paraphrased from source manuscript to ensure accuracy.

*AMSTAR* Assessment of Multiple Systematic Reviews, *NOAC* new oral anticoagulants, *VTE* venous thromboembolism, *THA* total hip arthroplasty, *TKA* total knee arthroplasty, *UKA* unicompartmental arthroplasty, *TLIF* transforaminal lumbar interbody fusion, *PLIF* posterior lumbar interbody fusion, *CEA* cost-effectiveness analysis, *CUA* cost-utility analyses, *TJA* total joint arthroplasty, *CVM* contingent-valuation, *QHES* quality of Health Economic Studies method.
