# Supplementary material for: Trends and Themes in the Study of Value in Orthopedic Surgery: A Systematic Review
Source: HSS J. 2023 Oct 24;21(1):93–101. doi: 10.1177/15563316231204040 (PMC11748386; doi:10.1177/15563316231204040)
Supplement: sj-docx-3-hss-10.1177_15563316231204040 – Supplemental material for Trends and Themes in the Study of Value in Orthopedic Surgery: A Systematic Review [file sj-docx-3-hss-10.1177_15563316231204040.docx]

**Supplemental Table 3** Study design

|  | **Arthroplasty** | **Foot and Ankle** | **Hand** | **Spine** | **Sports Medicine** | **Trauma** | **Upper Extremity** | **General/**  **Unspecified** | **All** |
| --- | --- | --- | --- | --- | --- | --- | --- | --- | --- |
|  |  |  |  |  |  |  |  |  |  |
| **Health Economic Study Design** |  |  |  |  |  |  |  |  |  |
| Cost-Utility Analysis | 61.1% | 67.5% | 100.0% | 41.3% | 73.4% | 34.7% | 98.5% | 37.8% | **55.0%** |
| Cost-Effectiveness Analysis | 19.3% | 27.5% | 0.0% | 42.5% | 21.9% | 2.7% | 0.0% | 2.7% | **23.7%** |
| Cost Description/Analysis | 11.8% | 0.0% | 0.0% | 2.8% | 0.0% | 37.3% | 1.5% | 0.0% | **8.6%** |
| Unclear | 0.3% | 0.0% | 0.0% | 10.2% | 0.0% | 22.7% | 0.0% | 0.0% | **5.3%** |
| Combination of Multiple Methods | 6.2% | 2.5% | 0.0% | 0.0% | 4.7% | 0.0% | 0.0% | 0.0% | **2.8%** |
| Contingent Valuation Study | 0.0% | 0.0% | 0.0% | 0.0% | 0.0% | 0.0% | 0.0% | 59.5% | **2.7%** |
| Cost-Minimization Analysis | 0.3% | 2.5% | 0.0% | 2.0% | 0.0% | 2.7% | 0.0% | 0.0% | **1.1%** |
| Cost-Benefit Analysis | 1.0% | 0.0% | 0.0% | 0.8% | 0.0% | 0.0% | 0.0% | 0.0% | **0.6%** |
| Cost Health Outcome | 0.0% | 0.0% | 0.0% | 0.4% | 0.0% | 0.0% | 0.0% | 0.0% | **0.1%** |
|  |  |  |  |  |  |  |  |  |  |
| **Perspective** | **Arthroplasty** | **Foot and Ankle** | **Hand** | **Spine** | **Sports Medicine** | **Trauma** | **Upper Extremity** | **General/**  **Unspecified** | **All** |
| Healthcare or Hospital System | 47.8% | 57.5% | 14.3% | 36.7% | 69.7% | 1.8% | 0.0% | 25.0% | **40.5%** |
| Societal | 20.7% | 15.0% | 7.1% | 30.6% | 18.2% | 10.9% | 0.0% | 50.0% | **23.5%** |
| Payer | 12.0% | 7.5% | 0.0% | 10.1% | 3.0% | 69.1% | 0.0% | 6.3% | **14.8%** |
| Unclear/Not Specified | 10.1% | 2.5% | 71.4% | 14.9% | 0.0% | 0.0% | 0.0% | 18.8% | **11.6%** |
| Multiple Perspectives | 7.2% | 17.5% | 7.1% | 6.9% | 6.1% | 18.2% | 0.0% | 0.0% | **8.4%** |
| Other | 2.2% | 0.0% | 0.0% | 0.8% | 3.0% | 0.0% | 0.0% | 0.0% | **1.3%** |
| Total |  |  |  |  |  |  |  |  |  |
|  |  |  |  |  |  |  |  |  |  |
| **Model- or Trial-Based** | **Arthroplasty** | **Foot and Ankle** | **Hand** | **Spine** | **Sports Medicine** | **Trauma** | **Upper Extremity** | **General/**  **Unspecified** | **All** |
| Trial-Based | 43.3% | 52.5% | 0.0% | 70.8% | 38.1% | 78.7% | 42.4% | 42.9% | **53.3%** |
| Model-Based | 50.2% | 47.5% | 28.6% | 10.7% | 47.4% | 21.3% | 57.6% | 57.1% | **36.0%** |
| Unclear/Unknown | 6.5% | 0.0% | 71.4% | 18.5% | 14.4% | 0.0% | 0.0% | 0.0% | **10.6%** |
|  |  |  |  |  |  |  |  |  |  |
| **Time Horizons** | **Arthroplasty** | **Foot and Ankle** | **Hand** | **Spine** | **Sports Medicine** | **Trauma** | **Upper Extremity** | **General/**  **Unspecified** | **All** |
| 0-5 | 28.2% | 0.0% | 71.4% | 77.8% | 16.5% | 30.7% | 0.0% | 33.3% | **39.4%** |
| 6-10 | 6.3% | 2.5% | 7.1% | 9.4% | 3.1% | 4.0% | 0.0% | 26.7% | **6.5%** |
| >10 | 10.3% | 2.5% | 14.3% | 1.7% | 5.2% | 4.0% | 0.0% | 26.7% | **6.3%** |
| Lifetime | 22.9% | 5.0% | 7.1% | 3.4% | 17.5% | 12.0% | 24.2% | 13.3% | **14.5%** |
| Other Breakdown | 6.6% | 0.0% | 0.0% | 0.4% | 0.0% | 0.0% | 75.8% | 0.0% | **5.7%** |
| Not Specified | 25.7% | 90.0% | 0.0% | 7.3% | 57.7% | 49.3% | 0.0% | 0.0% | **27.6%** |
