# Supplementary material for: Trends and Themes in the Study of Value in Orthopedic Surgery: A Systematic Review
Source: HSS J. 2023 Oct 24;21(1):93–101. doi: 10.1177/15563316231204040 (PMC11748386; doi:10.1177/15563316231204040)
Supplement: sj-docx-4-hss-10.1177_15563316231204040 – Supplemental material for Trends and Themes in the Study of Value in Orthopedic Surgery: A Systematic Review [file sj-docx-4-hss-10.1177_15563316231204040.docx]

**Supplemental Figure 1** Preferred reporting items for systematic reviews and meta-analysis (PRISMA) diagram.

**Supplemental Figure 2.** Quality assessment tools used by included studies. Two studies used both QHES and CHEERS evaluation tools. *QHES* Quality of Health Economic Studies, *CHEERS* Consolidated Health Economic Evaluation Reporting Standards, *CHEC* Consensus Health Economic Criteria, *CEA* Cost Effectiveness Analysis, *BMJ* British Medical Journal, *ISPOR* Professional Society for Health Economics and Outcomes Research formerly International Society for Pharmacoeconomics and Outcomes Research, *AdViSHE* Assessment of the Validation Status of Health-Economic decision models.

**Supplemental Tables**

**Supplemental Table 1.** The complete search strategies used to conduct a systematic review of published systematic reviews relating to economic analysis in orthopedic surgery.

**Supplemental Table 2** Study overview.

**Supplemental Table 3** Study design.
