# Supplementary figures and images for: Trends and Themes in the Study of Value in Orthopedic Surgery: A Systematic Review
Source: HSS J. 2023 Oct 24;21(1):93–101. doi: 10.1177/15563316231204040 (PMC11748386; doi:10.1177/15563316231204040)

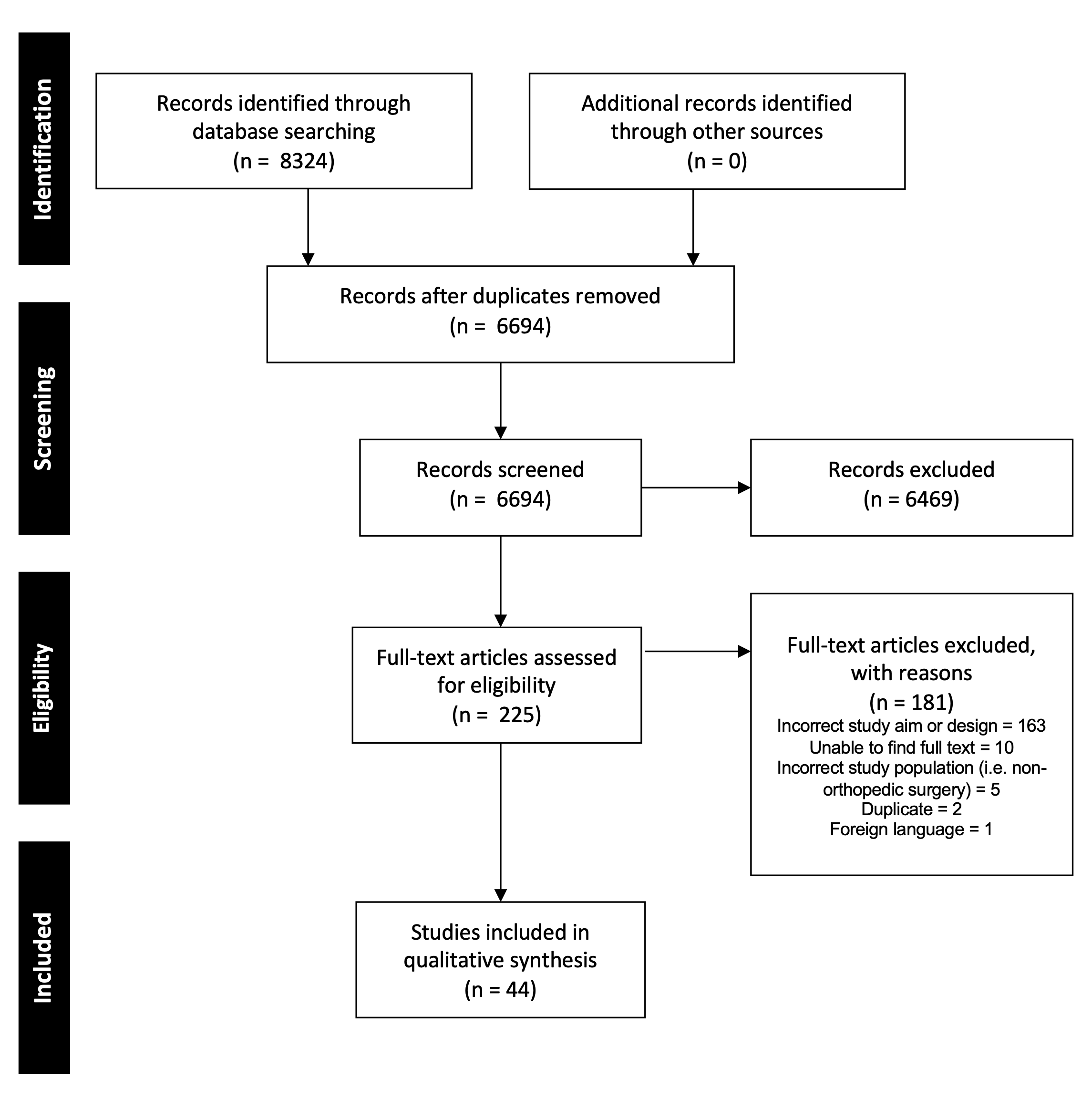

Supplement: sj-tiff-5-hss-10.1177_15563316231204040 – Supplemental material for Trends and Themes in the Study of Value in Orthopedic Surgery: A Systematic Review [file sj-tiff-5-hss-10.1177_15563316231204040.tiff]

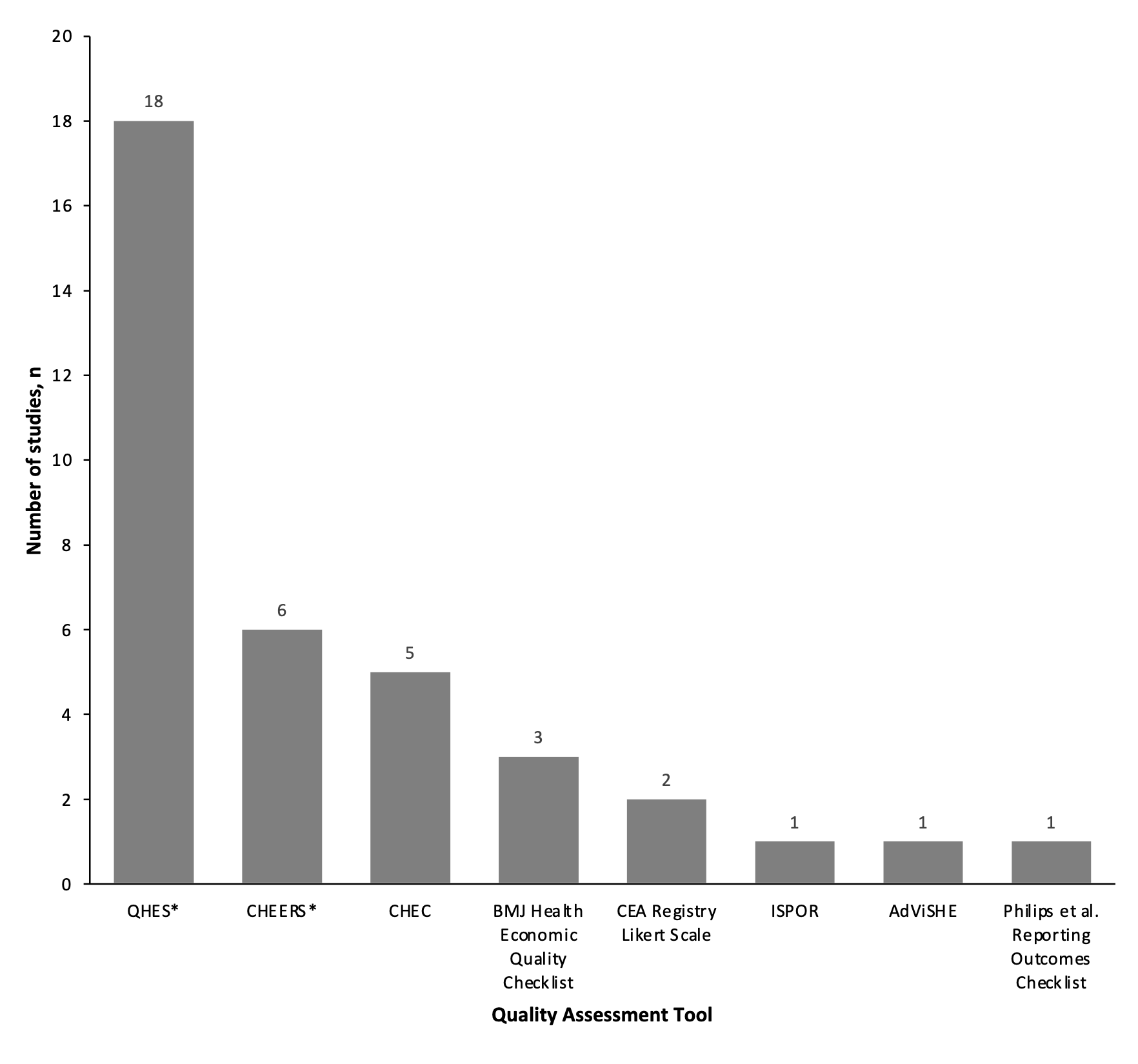

Supplement: sj-tiff-6-hss-10.1177_15563316231204040 – Supplemental material for Trends and Themes in the Study of Value in Orthopedic Surgery: A Systematic Review [file sj-tiff-6-hss-10.1177_15563316231204040.tiff]
